# Editorial on “Characteristics and risk profiles of patients with pulmonary arterial or chronic thromboembolic pulmonary hypertension living permanently at >2500 m of high altitude in Ecuador”

**DOI:** 10.1002/pul2.12428

**Published:** 2024-08-19

**Authors:** Samantha Sharma, Naresh Singh

**Affiliations:** ^1^ Indiana University Simon Comprehensive Cancer Center Indiana University School of Medicine Indianapolis Indiana USA; ^2^ Department of Medical and Molecular Genetics Indiana University School of Medicine Indianapolis Indiana USA

Pulmonary hypertension (PH) is a complex cardiovascular disorder characterized by elevated mean pulmonary artery pressure >20 mmHg at rest.^1^ PH is classified into five groups based on etiology, each with unique underlying causes and pathophysiological mechanisms: pulmonary arterial hypertension (PAH), PH due to left heart disease, PH due to lung disease or hypoxia, chronic thromboembolic pulmonary hypertension (CTEPH), and PH with unclear multifactorial mechanisms.^1^ The global prevalence of PH is estimated to be around 1%, with the majority of cases classified as PH associated with left heart or chronic lung disease while the prevalence of PAH and CTEPH is considerably lower, estimated at approximately 100 per million population.^2^ While the prevalence of PH is estimated to be similar worldwide, there are limited data on the characteristics and outcomes of PH patients living at high altitudes. Hypobaric hypoxia at high‐altitude imposes several genetic and physiological changes that help individuals living at various altitudes to maintain adequate oxygenation and function in the face of chronic hypoxia.^3^ However, the chronic hypoxic environment may exacerbate the occurrence and management of pulmonary vascular diseases (PVD).^4^

In this issue of Pulmonary Circulation, Hoyos et al. (2024) address this knowledge gap by performing a cross‐sectional study investigating the characteristics and risk profiles of patients with PAH or CTEPH living at high altitudes (>2500 m) in Ecuador.^5^ The study is notable for its focus on a unique and understudied population, as most research on PH has been conducted in low‐altitude populations, offering valuable insights into the impact of high altitude on PVD. The study's strengths lie in its detailed characterization of the patient cohort, including hemodynamic parameters, functional class, and exercise performance. The authors also provide an overview of the treatment strategies employed in this setting. The study found that despite living at high altitudes, these patients exhibit arterial oxygen levels similar to healthy newcomers at comparable altitudes and to PVD patients living at low altitudes.^5^ This finding challenges the traditional understanding of the impact of high altitude on PVD, which is typically associated with worsened hypoxemia and increased pulmonary vascular resistance.^6^ Interestingly, the study reveals that these patients have relatively low and stable risk profiles, as assessed by functional class, 6‐min walk distance, and NT‐pro‐brain natriuretic peptide levels, despite having severe hemodynamic compromise.^5^ This finding is consistent with previous research that has shown that individuals living at high altitudes can adapt to chronic hypoxia and maintain relatively normal exercise capacity.^4^

The findings of this study have possible implications for understanding the pathogenesis of PH in different populations and environments. The study suggests that current risk assessment models, which are primarily based on data from low‐altitude populations, may not be entirely applicable to high‐altitude dwellers.^5^ This highlights the need for further research to develop risk stratification tools and treatment strategies that are specifically tailored to high‐altitude populations. However, the small sample size (36 patients) dominated by female patient group limits the generalizability of these findings.^5^ Additionally, the cross‐sectional design precludes any conclusions about the long‐term outcomes of these patients, and further research, particularly longitudinal studies, are needed to confirm these findings and to investigate the long‐term effects of high altitude on PVD. Further, the authors acknowledge the potential for selection bias, as the study only included ambulatory patients who were able to visit the clinic. This may have excluded patients with more severe disease, potentially skewing the results. Despite these limitations, the study highlights the implications in the management of PVD in high‐altitude populations. The findings suggest that favorable outcomes are achievable for these patients, even in settings with limited resources. This is encouraging for healthcare providers working in high‐altitude regions, as it suggests that effective treatment strategies can be implemented even with limited resources and expensive combination therapies.

In conclusion, Hoyos et al. provide valuable insights into the characteristics and risk profiles of PVD patients living at high altitudes. This study underscores the importance of considering the unique physiological challenges faced by high‐altitude populations in the management of PH. This could open new avenues for research into the protective effects of hypoxia‐induced adaptations and their potential in therapeutic applications.

## AUTHOR CONTRIBUTIONS

Samantha Sharma and Naresh Singh conceived and contributed to writing the manuscript. Samantha Sharma and Naresh Singh contributed equally to this article.

## CONFLICT OF INTEREST STATEMENT

The author declares no conflict of interest.

## FUNDING INFORMATION

None.

## ETHICS STATEMENT

The authors have nothing to report.
